# To Measure or Not to Measure: Direct Oral Anticoagulant Laboratory Assay Monitoring in Clinical Practice

**DOI:** 10.1155/2023/9511499

**Published:** 2023-02-22

**Authors:** Tania Ahuja, Veronica Raco, Sharonlin Bhardwaj, David Green

**Affiliations:** ^1^NYU Lanogne Health, Department of Pharmacy, 550 First Avenue, New York, New York, 10016, USA; ^2^New York University Grossman School of Medicine, Department of Medicine, 550 First Avenue, New York 10016, USA; ^3^Olive-View UCLA Medical Center, 14445 Olive View Dr, Sylmar, CA 91342, USA

## Abstract

The need for therapeutic drug monitoring of direct oral anticoagulants (DOACs) remains an area of clinical equipoise. Although routine monitoring may be unnecessary given predictable pharmacokinetics in most patients, there may be altered pharmacokinetics in those with end organ dysfunction, such as those with renal impairment, or with concomitant interacting medications, at extremes of body weight or age, or in those with thromboembolic events in atypical locations. We aimed to assess real-world practices in situations in which DOAC drug-level monitoring was used at a large academic medical center. A retrospective review of the records of patients who had a DOAC drug-specific activity level checked from 2016 to 2019 was included. A total of 119 patients had 144 DOAC measurements (apixaban (*n* = 62) and rivaroxaban (*n* = 57)). Drug-specific calibrated DOAC levels were within an expected therapeutic range for 110 levels(76%), with 21 levels (15%) above the expected range and 13 levels (9%) below the expected range. The DOAC levels were checked in the setting of an urgent or emergent procedure in 28 patients (24%), followed by renal failure in 17 patients (14%), a bleeding event in 11 patients (9%), concern for recurrent thromboembolism in 10 patients (8%), thrombophilia in 9 patients (8%), a history of recurrent thromboembolism in 6 patients (5%), extremes of body weight in 7 patients (5%), and unknown reasons in 7 patients (5%). Clinical decision making was infrequently affected by the DOAC monitoring. Therapeutic drug monitoring with DOACs may help predict bleeding events in elderly patients, those with impaired renal function, and in the event of an emergent or urgent procedure. Future studies are needed to target the select patient-specific scenarios where monitoring DOAC levels may impact clinical outcomes.

## 1. Introduction

The direct oral anticoagulants (DOACs) have become preferred over vitamin K antagonists (VKAs), such as warfarin, for stroke prevention in atrial fibrillation (AFib) and also for the prevention and treatment of venous thromboembolism (VTE) [1–9]. The DOACs consist of the factor Xa inhibitors, apixaban, edoxaban, and rivaroxaban and the direct thrombin inhibitor, dabigatran. In contrast to VKA, the DOACs do not require routine laboratory monitoring, and have fewer drug-drug interactions, limited drug-food interactions, and predictable pharmacokinetics in most patients [1–9]. Notably, although drug-level monitoring is not routinely required, many cohorts of patients that may benefit from a DOAC over a VKA were excluded from the pivotal landmark trials, including those with impaired renal function, extremes of body weight, solid organ transplants, and active cancer [1–9]. Since their approval, there has been interest in monitoring drug levels to better predict bleeding and thromboembolic risk in select cohorts [10]. National guidelines, such as those of the International Society of Hemostasis and Thrombosis (ISTH) and the National Comprehensive Cancer Network (NCCN), now recommend DOACs for cancer-associated VTE after registry data and noninferiority trials found DOACs to be comparable to those who received low molecular weight heparin (LMWH) [11, 12]. However, there remain clinical scenarios where a DOAC activity level may assist in clinical decision making, such as when suspecting under- or over-coagulation, in preparation for a surgical procedure, or in the setting of bleeding or the need for thrombolysis.

Since global coagulation laboratory parameters, such as the activated partial thromboplastin time (aPTT), partial thromboplastin time (PT), and international normalized ratio (INR) are not calibrated to assess the anticoagulant effects of DOACs, these are not preferred to assess the safety or efficacy of the DOACs. There remains limited data correlating DOAC levels to bleeding, thromboembolic events, or overall mortality [13, 14]. Given the potential for altered pharmacodynamic effects in those with hepatic, renal, or cardiac dysfunction, extremes of body weight or age, and/or thrombophilia, a hematologist may often be consulted on cases where the use of a calibrated drug assay may assist in the selection of the dose of anticoagulation. Still, there remains limited evidence to support this, and the optimal therapeutic ranges vary based on assay, type of DOAC, and dose of DOAC [15–18]. Further, the need for a DOAC-calibrated drug assay may assist with a clinical decision regarding anticoagulation reversal in the event of bleeding, the need for thrombolysis for an ischemic stroke, or for other emergent procedures [12, 19–22]. At New York University Langone Health, a large academic medical center, antithrombotic stewardship focusing on selection of anticoagulation, dosing, and monitoring is embedded into clinical decision-making tools available in the electronic medical record. Subject matter content is reviewed through the Antithrombotic and Hemostatic Therapy Oversight Group (ATHOG), consisting of interdisciplinary involvement from physicians in hematology, cardiology, neurology, vascular surgery, and interventional radiology, along with clinical pharmacotherapy specialists, nursing, and hematology laboratory representatives. In September of 2016, DOAC laboratory assays calibrated to drug levels were made available. Given the limited data published to support dose adjustments, ATHOG provided guidance that was communicated to committee members but did not formally implement a guideline for which clinical scenarios to check levels in, or how to correlate them with bleeding or thromboembolic events. The aim of our retrospective review was to determine the indications for where clinicians checked these levels and how the levels assisted in the overall management of patients on a DOAC.

## 2. Methods

This was a single-centered, institutional review board (IRB)-approved, retrospective cohort study at NYULH of adult patients prescribed a DOAC with a corresponding DOAC drug-specific level from September 2016 to January 2019. These patients were included if they were at least 18 years of age and received anticoagulation with a DOAC, including dabigatran, apixaban, or rivaroxaban. Since edoxaban was not available on hospital formularies, we did not have DOAC drug-level assessments available for this drug.

Data collection consisted of a retrospective review of baseline demographics, medical history, surgical history, or trauma within 6 months of anticoagulation initiation, characteristics of thromboembolic risk factors or bleeding risk factors, and DOAC initiation. Risk factors for thromboembolism included documentation of prolonged immobility, cancer history, known trauma or surgery, or known thrombophilia. Other data collected included dosing of DOAC, indication for DOAC, timing of administration of DOAC and timing of DOAC laboratory assay, concomitant potential drug interactions, including antiplatelet therapy, and history of bleeding or thromboembolic events while on DOAC therapy. In order to characterize whether the DOAC-specific level was a peak value or trough value, electronic medical records were reviewed for documentation of intent for peak (2–4 hours for apixaban or rivaroxaban and 2 hours for dabigatran) or trough (before next dose) or a random sample, as documented in notes by the ordering physician along with timing of laboratory result. If a corresponding note did not specify the intent, DOAC-specific levels were characterized by the date/time of the sample compared to the documentation of the administration time of the DOAC. In the absence of documentation or known timing of administration of DOAC, samples were characterized as random sampling. Measured DOAC levels were compared to expected steady-state ranges of DOAC levels based on previously published data. Data were managed utilizing Research Electronic Data Capture (REDCap), a secure informatics system designed to support data collection across various research disciplines [23].

The primary outcome was the percentage of DOAC assay levels checked based on clinical indication. Secondary outcomes include the percentage of levels within an expected therapeutic range and whether testing affected clinical change in antithrombotic management. In addition, the safety and efficacy of antithrombotic therapy were evaluated. Safety was assessed by the presence of any bleeding events while on antithrombotic therapy. Major bleeding events were defined using the International Society of Thrombosis and Hemostasis criteria: fatal bleeding, and/or symptomatic bleeding in a critical area or organ (including intracranial, intraspinal, intraocular, retroperitoneal, intraarticular, pericardial, or intramuscular bleeding with compartment syndrome), and/or a greater than 2 g/dl decrease in hemoglobin or bleeding necessitating 2 or 3 units of whole blood or packed red blood cells [24]. Clinically relevant minor bleeding was defined as overt bleeding noted by a physician that was not attributed to an alternative source. Efficacy was assessed by the lack of any thromboembolic events, including stroke or any VTE, while on therapy with a DOAC. In addition, if changes in DOAC therapy occurred, or antithrombotic therapy was discontinued, the rationale for discontinuations were assessed. Reasons for the discontinuation was included bleeding events, thrombocytopenia, acute kidney injury (AKI), defined by the RIFLE criteria, or not classified [25].

Venous blood samples were drawn to assess DOAC concentrations. Dabigatran levels were available for testing using the HemosIL DTI assay. Apixaban and rivaroxaban levels were measured using the chromogenic HemosIL liquid anti-Xa assay (Instrumentation Laboratory, United States). All assays were measured on an ACL TOP 500 coagulometer (Instrumentation Laboratory, United States).

Patient characteristics were described as proportions for categorical variables and as medians and interquartile ranges for continuous variables without a normal distribution. The coefficient of variation was calculated for all DOAC levels based on expected therapeutic ranges and renal function. Data were analyzed using the Software Package for Statistics and Simulation (IBM SPSS version 22, IMB Corp. Armonk, NY).

## 3. Results

### 3.1. Patient Characteristics

In total, 119 patients underwent 144 DOAC measurements [apixaban (*n* = 62) and rivaroxaban (*n* = 57), with no dabigatran levels checked] during the study period.  Table 1 presents the demographics and clinical characteristics of the patients that had DOAC levels measured (50% female), median age 74 (27–102) years, with 14% of the cohort having a body mass index (BMI) greater than 35 and 28% of the cohort having renal impairment at the time of DOAC testing. Baseline characteristics did not differ according to DOAC. The majority of DOAC levels occurred during acute hospitalization (68, 58%), with the remainder in outpatient clinics. The clinical referring service for DOAC monitoring in the outpatient setting was predominantly hematology (40, 78%). Indications for anticoagulation therapy were the treatment of VTE in 59 (50%) followed by stroke prevention with AF in 46 (39%).

### 3.2. Primary Outcome: Indications for DOAC Level Monitoring

The DOAC levels were checked in the setting of an urgent or emergent procedure in 38 patients (32%), followed by renal failure in 17 patients (14%), a bleeding event in 11 patients (9%), concern for recurrent thromboembolism in 10 patients (8%), thrombophilia in 9 patients (8%), a history of recurrent thromboembolism in 6 patients (5%), and extremes of body weight in 21 patients (18%). There were 7 patients (20%) who did not have a clear indication for DOAC level measurement documented. The breakdown of DOAC levels checked by apixaban or rivaroxaban can be found in Table 2.

### 3.3. Secondary Outcomes: DOAC Drug Levels and Clinical Management

DOAC drug levels were within the expected range for 110 levels(76%), with 21 levels (14.5%) above the expected range and 13 levels (9%) below the expected range. The type of DOAC used, gender, and setting of testing did not differ according to the drug-level result. There were a total of 16 peaks and 23 troughs checked for rivaroxaban 20 mg daily, with a median value of 315.1 ng/mL (19–517 ng/mL) and 98 ng/mL (14–209 ng/mL), respectively. There were a total of 25 peaks and 19 troughs checked for apixaban, with 11 peaks and 11 troughs with the 2.5 mg twice daily regimen and 14 peaks and 8 troughs for the 5 mg twice daily regimen. The median apixaban peak values were 259 ng/mL (102–657 ng/mL) and 255 ng/mL (46–477 ng/mL) for the 2.5 mg and 5 mg doses, respectively. The median apixaban trough values were 105 ng/mL (41–274 ng/mL) and 91 ng/mL (5–173 ng/mL) for the 2.5 mg and 5 mg doses, respectively. The remaining 61 levels were classified as random sampling in the setting of bleeding event or need for urgent procedure or unknown reasons.

**Figure 1:**
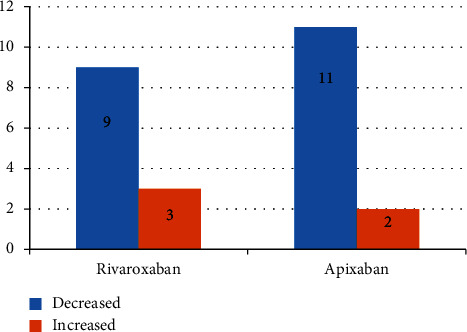
Change in DOAC dose.

Clinical decisions regarding DOAC management, including dose adjustments, changes in antithrombotic therapy, and/or discontinuations as a result of DOAC monitoring, occurred in 39 (33%) of the measurements performed. This resulted in a decrease in DOAC dose (*n* = 20), an increase in DOAC dose (*n* = 5), the enabling of a procedure (*n* = 22), and the cancellation of a procedure (*n* = 1). A breakdowxn of changes in DOAC dose can be found in Figure 1.

### 3.4. Secondary Outcomes: Recurrent Thromboembolic Events and Bleeding

There were 15 patients who had thromboembolic events during DOAC therapy. Out of these, 9 were recurrent VTEs and 6 were ischemic strokes. In addition, 3 of these 15 patients had a correspondingly below-level DOAC level at the time of the event, including 1 patient with antiphospholipid syndrome. There were 19 patients (16%) that experienced a bleeding event during treatment with a DOAC, with 4 bleeds at a critical site, including 3 intracranial bleeds. For the intracranial bleeds, 2 random DOAC levels assessed around the time of the bleed were above the desired therapeutic range. The remaining 15 bleeding events were classified as clinically overt, with 6 patients on concomitant antiplatelet therapy. Out of the 19 bleeds, 13 patients had impaired renal function, defined as a creatinine clearance (CrCL) less than 60 mL/min at the time of the bleeding event. For patients that had a DOAC level checked in the setting of an emergent or urgent procedure (*n* = 14), 4 patients experienced bleed events, of which 3 occurred on apixaban and 1 on rivaroxaban. All procedures occurred emergently, within 12–24 hours after the DOAC was held, with levels below the desired therapeutic range. There was once a procedure that was cancelled. Further breakdown of ranges of levels and percentage of levels within and out of range can be found in the supplementary files in Supplementary Tables 1 and 2 and DOAC levels plotted according to time, dose and renal function can be found in Supplementary Figures 1–4.

## 4. Discussion

This retrospective study evaluated the clinical utility of DOAC level monitoring at a large academic medical center. We found DOAC levels had implications for clinical decision-making in the setting of impaired renal function, elderly patients, and those needing emergent or urgent procedures. However, routine DOAC level monitoring may be unnecessary, as clinical decision-making was infrequently affected by these results in our cohort. Our data highlight that most DOAC levels were within an expected therapeutic range, consistent with effects desired in those with VTE, AF, or cancer-associated thrombosis (CAT) [3–9, 11]. Still, in unique circumstances, knowledge of the DOAC drug level may help better predict bleeding or thrombosis [26–30]. Although there are no official recommendations to advise when DOAC drug levels may be best utilized, our data and evidence to date suggest that bleed risk may be better predicted in these circumstances [26–30].

We observed bleeding events most commonly in the inpatient setting in those with renal impairment, suggesting the relationship of elevated DOAC drug concentrations in this setting. This may be a case of correlation rather than causation, and the knowledge of such levels may have little impact on clinical care, with the exception of consideration for hemostatic agents, such as the use of prothrombin complex concentrates or other agents for reversal, such as andexanet alfa.

Older age has been demonstrated to be a risk factor for having a DOAC level above the expected range [26, 28, 31]. Notably, the median age for our cohort was 74 years, much older than the patients enrolled in the landmark trials [6–9]. Elderly patients are at increased risk of thromboembolic events due to vascular calcification and also bleeding events as hepatic and renal function decline with age, making antithrombotic therapy a double-edged sword [26, 28]. Future studies evaluating DOAC drug levels in elderly patients specifically may help guide clinical decision-making in this cohort.

Lastly, we found DOAC drug levels checked in the setting of urgent or emergent procedures. We observed bleeding most commonly when the procedure was not delayed more than 24 hours from the last known dose of the DOAC, suggesting that the DOAC drug-level knowledge did not result in a delay in the emergent procedure. Still, knowledge at this level may help clinicians anticipate bleeding and have hemostatic agents available as necessary. Even though cutoffs for peak and trough values for apixaban and rivaroxaban have been provided from the landmark trials, it is unknown if these trough values are considered safe to prevent procedural-related bleeding [21, 32–34]. Though preprocedure DOAC troughs of less than 30 ng/mL have been considered safe to proceed without an impact on bleed risk, timing from the last dose may better predict actual bleed risk [21, 34]. In the PAUSE trial, DOACs were interrupted for 24 hours for low bleed-risk procedures and 48 hours for high bleed-risk procedures [33]. However, with emergent procedures, 24–48 hours may be too long, and preprocedure DOAC levels may better help predict postprocedure bleed risk [29].

## 5. Conclusion

DOAC-calibrated assays to measure plasma concentrations are often limited by availability, lack of rapid turnaround, and lack of guidelines for clinical management of dose based on level. In addition, data correlating drug levels to clinical outcomes, such as recurrent thromboembolism or bleeding events remains limited. We observed the greatest use of these drug levels in certain clinical scenarios for acutely hospitalized patients at high risk for bleeding due to old age or renal dysfunction. Future studies are necessary in these specific clinical scenarios or when there may be concern for unpredictable pharmacokinetics to better predict the safety and tolerability of a DOAC. In addition, for those patients presenting to the hospital with bleeding, a DOAC-specific level may help predict the need for hemostatic or reversal agents. As DOACs have overtaken warfarin as the preferred oral anticoagulant for most patients, even traditional warfarin centric clinics now offer services to optimize patient selection for DOACsand assist with temporary interruption recommendations for procedures, including measurements of DOAC-specific levels in some circumstances [34].

Further, assessment of DOAC-calibrated therapeutic drug monitoring may be beneficial in cohorts not represented in landmark trials, as real-world prescribing has demonstrated that DOACs are often used off-label prior to trials to support use, such as in those with cancer [35]. Still, as we observed the majority of DOAC drug levels to be within an expected range with little impact on clinical outcomes, we suggest these levels be used cautiously and judiciously.

## Figures and Tables

**Table 1:** Patient baseline characteristics.

	Overall *N* = 119	Apixaban *n* = 62	Rivaroxaban *n* = 57
Age, years: median (IQR)	74 (27–102)	78 (33–102)	69 (27–91)
<65 years	37 (31)	13 (21)	24 (42)
65–74 years	26 (22)	11 (18)	15 (26)
≥75 years	56 (47)	38 (61)	18 (32)
Sex			
Male	60 (50)	31 (50)	29 (51)
Female	59 (50)	31 (50)	28 (49)
Body Mass index, kg/m^2^: median (IQR)	26.8 (17–64)	26.0 (17–49)	26.9 (17–64)
≥35	16 (13)	6 (10)	10 (18)
30–34.9	18 (15)	11 (18)	7 (12)
25–29.9	43 (36)	22 (35)	21 (37)
18.5–24.9	33 (28)	17 (27)	16 (28)
<18.5	4 (3)	3 (5)	1 (2)
Unknown	5 (4)	3 (5)	2 (3)
Past medical history			
Gastrointestinal bleed	8 (7)	7 (11)	1 (2)
Stroke/TIA	28 (24)	18 (29)	10 (18)
eGFR <60 mL/min	33 (28)	26 (42)	7 (12)
Diabetes	27 (23)	18 (29)	9 (16)
Indication for anticoagulant			
Stroke prevention with atrial fibrillation	49 (41)	14 (12)	35 (29)
Acute PE	28 (24)	16 (28)	12 (19)
Chronic PE	14 (12)	12 (21)	2 (3)
Acute DVT	31 (26)	18 (32)	13 (21)
Chronic DVT	24 (20)	19 (33)	5 (8)
Other	9 (8)	4 (7)	5 (8)

**Table 2:** Indication for laboratory assessment of DOAC level.

	Overall *N* = 119	Apixaban *n* = 62	Rivaroxaban *n* = 57
Urgent/emergent procedure	38 (32)	28 (45)	10 (18)
Acute kidney injury	17 (14)	10 (16)	7 (12)
Bleed event on DOAC	11 (9)	5 (8)	6 (11)
Recurrent VTE on DOAC	10 (8)	5 (8)	5 (9)
Thrombophilia	9 (8)	0 (0)	9 (16)
Other	7 (6)	2 (3)	5 (9)
History of recurrent VTE pre-DOAC	6 (5)	0 (0)	6 (11)
High body weight	16 (13)	10 (16)	6 (11)
Low body weight	5 (4)	2 (3)	3 (5)

## Data Availability

The data used to support the findings of this study are available from the corresponding author upon request.

## Additional Points

The need for routine DOAC-calibrated drugs levels is likely unnecessary. In real-world prescribing of DOACs, we observed drug levels monitored in certain clinical scenarios, specifically in elderly patients, those with renal impairment, or those needing urgent or emergent procedures. Future studies are necessary to establish associations between drug levels and clinical outcomes.

## Supplementary Materials

Supplementary Materials Table 1: Mean, median, and range of levels. Table 2: Percentage of levels in and out of range. Figure 1: Rivaroxaban levels with a 20 mg daily dose. Figure 2: Apixaban levels in all doses. Figure 3: Apixaban DOAC levels plotted against renal function. Figure 4: Rivaroxaban DOAC levels plotted against renal function.
