# Supplementary material for: To Measure or Not to Measure: Direct Oral Anticoagulant Laboratory Assay Monitoring in Clinical Practice
Source: Adv Hematol. 2023 Feb 22;2023:9511499. doi: 10.1155/2023/9511499 (PMC9977549; doi:10.1155/2023/9511499)
Supplement: Supplementary Materials — Table 1: Mean, median, and range of levels. Table 2: Percentage of levels in and out of range. Figure 1: Rivaroxaban levels with a 20 mg daily dose. Figure 2: Apixaban levels in all doses. Figure 3: Apixaban DOAC levels plotted against renal function. Figure 4: Rivaroxaban DOAC levels plotted against renal function. [file 9511499.f1.zip › SuppFigAdvHeme (1).docx]

Supplementary Figures 1 and 2: DOAC levels according to time and dose.

Supplementary Figures 3 and 4: DOAC levels according to renal function

INDEX A:

RIVAROXABAN Expected Levels:
                Trough Level    Peak Level
   20 mg daily 3-153 ng/mL     182-408 ng/mL
   15 mg daily 2-161 ng/mL     180-408 ng/mL
   10 mg daily 1-38 ng/mL      91-196 ng/mL
 This test was developed and its performance characteristics
 determined by the NYULMC Clinical Laboratories. It has not been
 cleared or approved by the US Food and Drug Administration (FDA).
 The FDA has determined that such clearance or approval is not
 necessary. The results of this test should be used in conjunction
 with clinical findings.
Performed by NYU Hospital Center Clinical Labs, 560 First Avenue, New
York, NY 10016. Director: Mark S. Lifshitz MD.

APIXABAN Expected Levels:
         Trough Level    41-230 ng/mL
         Peak Level      91-321 ng/mL
 This test was developed and its performance characteristics
 determined by the NYULMC Clinical Laboratories. It has not been
 cleared or approved by the US Food and Drug Administration (FDA).
 The FDA has determined that such clearance or approval is not
 necessary. The results of this test should be used in conjunction
 with clinical findings.
Performed by NYU Langone Hospitals, Tisch Hospital Clinical Labs. 560
First Avenue, NY, NY, 10016. Director: Maria E. Aguero Rosenfeld MD.

Figure 1: Rivaroxaban levels with 20mg daily dose


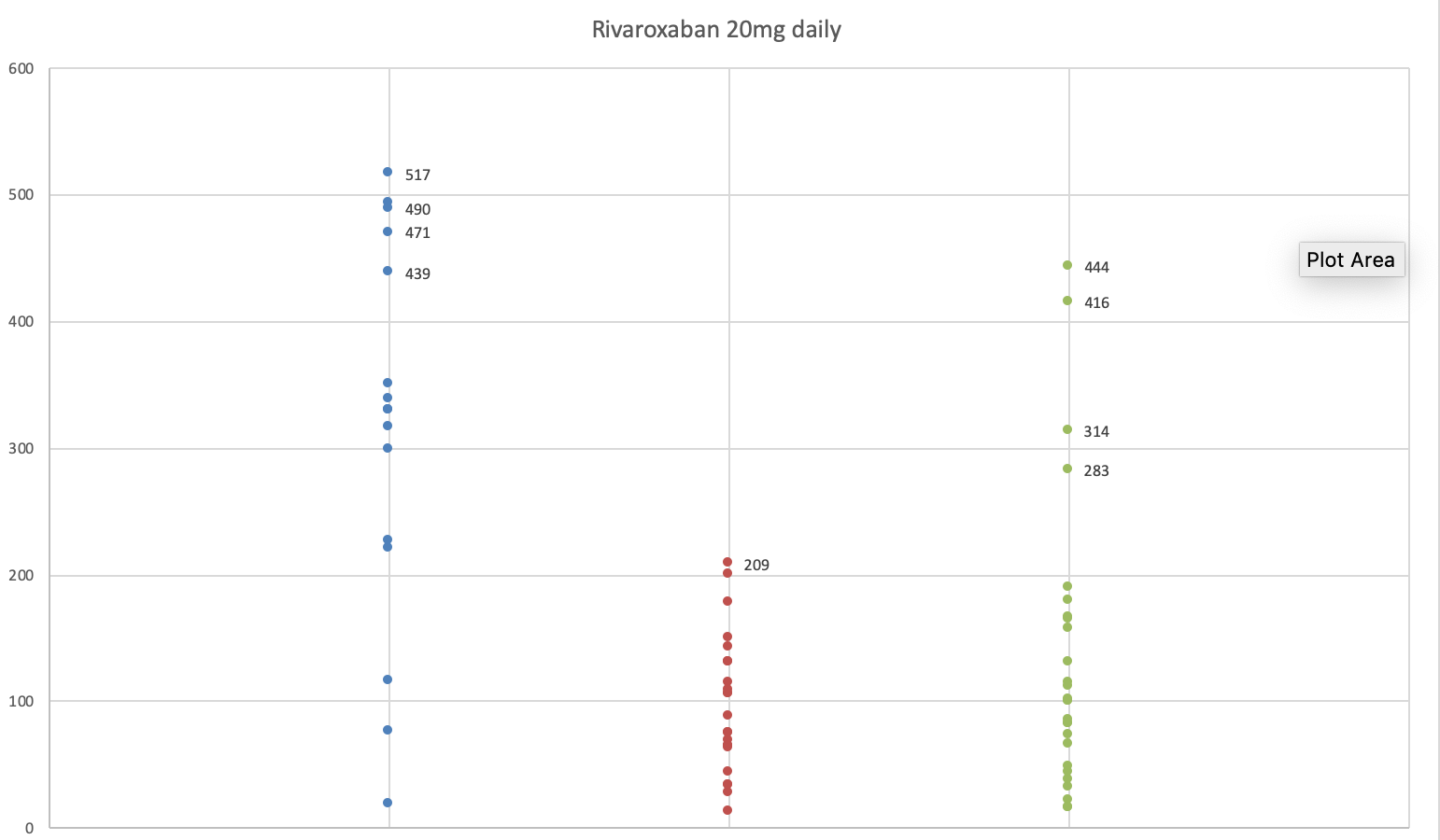
 Peak Trough Random

Figure 2: Apixaban levels in all doses


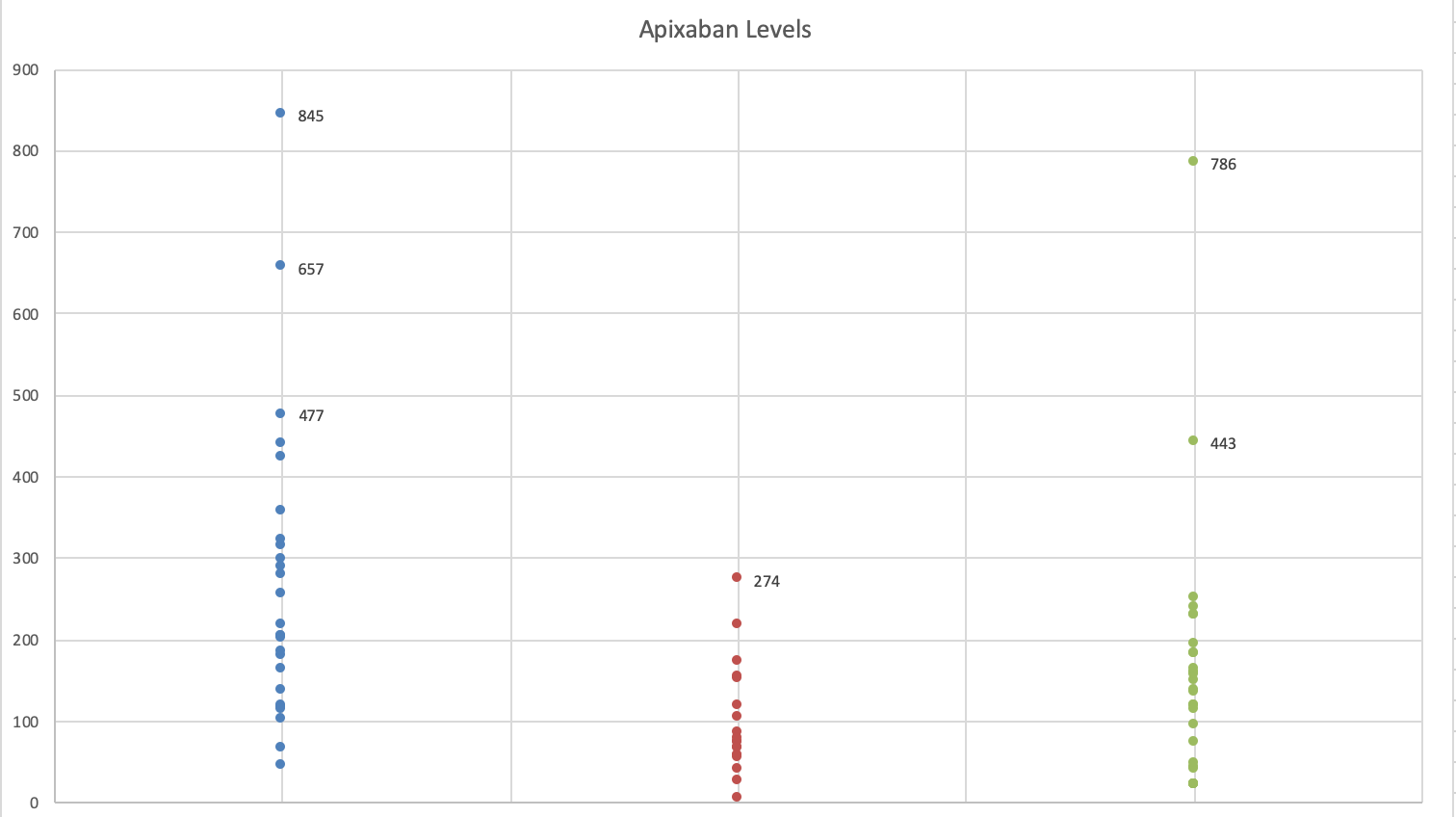
 Peak Trough Random

Figures 3: Apixaban DOAC levels plotted against renal function

Figures 4: Rivaroxaban DOAC levels plotted against renal function
