# Supplementary material for: To Measure or Not to Measure: Direct Oral Anticoagulant Laboratory Assay Monitoring in Clinical Practice
Source: Adv Hematol. 2023 Feb 22;2023:9511499. doi: 10.1155/2023/9511499 (PMC9977549; doi:10.1155/2023/9511499)
Supplement: Supplementary Materials — Table 1: Mean, median, and range of levels. Table 2: Percentage of levels in and out of range. Figure 1: Rivaroxaban levels with a 20 mg daily dose. Figure 2: Apixaban levels in all doses. Figure 3: Apixaban DOAC levels plotted against renal function. Figure 4: Rivaroxaban DOAC levels plotted against renal function. [file 9511499.f1.zip › Supplementary TablesAdvHeme.docx]

Supplementary Tables

Table 1: Mean, Median, Range of Levels

|  | Total No. Levels | MEAN VALUE | MEDIAN VALUE | RANGE |
| --- | --- | --- | --- | --- |
| PEAK Rivaroxaban  (20mg daily) | 16 | 315.1 | 330.5 | 19-517 |
| TROUGH Rivaroxaban  (20mg daily) | 23 | 97.5 | 89 | 14-209 |
| PEAK Apixaban  (2.5mg twice daily) | 11 | 259 | 218 | 102-657 |
| PEAK Apixaban  (5mg twice daily) | 14 | 254.9 | 247.5 | 46-477 |
| TROUGH Apixaban  (2.5mg twice daily) | 11 | 105.4 | 73 | 41-274 |
| TROUGH Apixaban (5mg twice daily) | 8 | 91.1 | 91.5 | 5-173 |
| RANDOM Rivaroxaban (20mg daily) | 27 | 133.1 | 100 | 16-444 |
| RANDOM Apixaban (2.5mg twice daily) | 11 | 105.5 | 136 | 22-183 |
| RANDOM Apixaban (5mg twice daily) | 21 | 174.9 | 149 | 22-786 |

Table 2: Percentage of Levels In and Out of Range

| **RIVAROXABAN** | **IN RANGE** | **NOT IN RANGE** | **POSITION OUT OF RANGE** |
| --- | --- | --- | --- |
| Peak levels | 8 | 8 | ABOVE – 5 , BELOW – 3 |
| Trough levels | 20 | 3 | ABOVE – 3, BELOW – 0 |
| Random levels | 21 | 6 | ABOVE – 2 , BETWEEN – 4 |
| **Total** | **49 (74)** | **17 (26)** | **ABOVE – 10 (59), BELOW – 3 (18), BETWEEN – 4 (24)** |
| **APIXABAN** |  |  |  |
| Peak levels  2.5mg  5mg  10mg  **Total** | 9  7  1  **17 (63)** | 2  7  1  **10 (37)** | ABOVE – 2  ABOVE – 5 , BELOW – 2  ABOVE – 1  **ABOVE – 8 (80), BELOW – 2 (20)** |
| Trough levels  2.5mg  5mg  **Total** | 10  6  **16 (84)** | 1  2  **3 (16)** | ABOVE – 1  BELOW – 2  **ABOVE – 1 (33), BELOW – 2 (66)** |
| Random levels  2.5mg  5mg  **Total** | 9  19  **28 (88)** | 2  2  **4 (12)** | BELOW – 2  ABOVE – 2  **ABOVE – 2 (50), BELOW – 2 (50)** |
| **TOTAL ALL LEVELS** | **110 (76)** | **34 (24)** | **ABOVE – 21 (62)**  **BELOW – 9 (26)**  **BETWEEN – 4 (12)** |

*Value in parentheses represents percentage of total in category
